# DAILY STRESS CONTRIBUTES TO GENDER DISPARITIES IN LONG-TERM CHRONIC DISEASE RISK

**DOI:** 10.1093/geroni/igad104.1763

**Published:** 2023-12-21

**Authors:** Kelly Cichy, Athena Koumoutzis, Dakota Witzel, Robert Stawski

**Affiliations:** Kent State University, Kent, Ohio, United States; Miami University, Oxford, Ohio, United States; Penn State University, State College, Pennsylvania, United States; University of Essex, Colchester, England, United Kingdom

## Abstract

Compared to men, women exhibit worse health outcomes, including increased risk for chronic disease (Carmel, 2019). Exposure and affective responses to daily stressors represent a risk factor for long-term health (Piazza et al., 2013) with research revealing a complex pattern of gender differences in affective responses to stressors (Birditt et al., 2005; Witzel et al. 2022). Together, findings suggest stressor exposure and affective reactivity may represent accessible intervention targets to reduce chronic disease risk and health disparities. Using data from Wave II of the National Study of Daily Experiences and MIDUS Waves II and III, this study explores: 1) gender differences in chronic disease risk; 2) prospective effects of differential exposure and reactivity on chronic disease risk 10 years later; and 3) whether daily stress processes (DSP) partially account for gender differences in chronic disease. Respondents include adults ages 34-84 years (N = 1209), who reported on daily stressors and affect for eight days during NSDE II and on chronic conditions during MIDUS III. Covariate-adjusted multilevel models revealed women reported more chronic conditions compared to men (p < .0001). DSP processes, including differential exposure (p < .01) and affective reactivity (p < .05) are associated with increased chronic conditions. Further, the magnitude of the gender differences in chronic conditions was reduced after accounting for DSP variables. Findings highlight the contribution of DSP for chronic disease risk and gender disparities in physical health. Implications for addressing DSP as accessible intervention targets will be discussed.

